# Notes on a Case of Variola Nigra. (Malignant or Black Smallpox.)

**Published:** 1902-03

**Authors:** D. S. Davies

**Affiliations:** Medical Officer of Health for Bristol City and Port


### NOTES ON A CASE OF VARIOLA NIGRA.

### (MALIGNANT OR BLACK SMALLPOX.)

BY

D. S. Davies, M.D.,

*Medical Officer of Health for Bristol City and Port.*

---

The patient, a strong, able-bodied seaman, came from Boston, Mass., to Portland, Maine, U.S.A., where he signed on a steamer bound for Avonmouth, Bristol, on 21st December, 1901, arriving in Bristol on Monday, 6th January, 1902. Smallpox was prevalent in Boston between July and December, 1901: 344 cases and 41 deaths were recorded during that period. In February, 1902, two further cases of smallpox were introduced into Bristol, and several cases into Liverpool from this part of the United States.

On January 4th, fourteen days after joining the vessel and two days before arrival, he was definitely ill, having probably been sickening for a day previously; he then complained of general chilliness and considerable pains in the sacral region, which he attributed to exposure and strain during rough weather.

On arrival, he applied at the General Hospital on January 6th, having reached the Hospital by train and cab, and was seen by Dr. Symes, who, recognising the gravity of the case, at once reported it, and the patient was admitted on the same evening to one of the city hospitals, under the care of Mr. Heaven, with whom I saw him in consultation on the following morning.

The patient's temperature was 100° F., which was not exceeded during the observed course of his illness, and he presented the characteristic clinical picture of this fortunately rare and always fatal form of smallpox.

He was obviously very ill, complaining of pain in the sacral region, but chiefly of abdominal pain; the tongue and mouth dry and brown, the saliva blood-stained, the face flushed and puffy, but with no sign of any eruption, which is characteristically suppressed in this variety of smallpox. The body showed, however, an almost universal dusky-red flush, more marked in the axillæ and groins; numerous small petechial spots were present on the trunk and limbs, with more marked and larger bluish-black hemorrhages on the lower part of the abdomen, in the groins, and on the upper part of the inner aspect of the thighs.

On the right thigh were a few abortive vesicles, and one shotty papule on the left arm, but nothing in the shape of a defined smallpox eruption; the pulse was fairly regular and full, the mental condition clear, questions being readily and sensibly answered.

The left conjunctiva showed the characteristic hemorrhage, forming a continuous clot over the outer half of the conjunctiva; the right was deeply congested, but showed no continuous hemorrhage.

The urine was scanty and "coffee coloured;" a few ounces were passed three times in the twenty-four hours.

There was frequent vomiting, chiefly of food administered, with some clots of blood.

He continued much in the same condition for twenty-four hours after admission, when he was suddenly seized about eight o'clock on the 7th January with a convulsive attack affecting the face and arms, which lasted about ten minutes, and during which he died.

An attempt was made to relieve the abdominal pain and vomiting by nepenthe and bismuth. The bowels were relieved by enema, which brought away ordinary fæces; they were relieved naturally twice afterwards; the motions passed were light coloured, with no evidence of bleeding.

The man's age was 29; he showed two scars of infant vaccination: he was therefore what Dr. Bond has called "de-vaccinated," having outlived the term of protection from primary vaccination.

Dr. Symes has kindly informed me that when first seen his condition was pretty much as I have described, but there was then no conjunctival hemorrhage; the general dusky-red flush, which showed "bathing drawers" distribution, had not been succeeded by the petechial spots and hemorrhages. The temperature was 100° F., pulse 120. Heart and lungs normal, no enlargement of liver or spleen noticeable. The pupils normal in reaction to light and accommodation, and no squint. Beyond a slight haziness as to dates, the patient was mentally quite clear.

This invariably fatal form of smallpox, which is hemorrhagic from the commencement, with an entire suppression of the characteristic eruption, must not be confused with the hemorrhagic conditions which may come on during the development of the true smallpox eruption, either at the papular, vesicular, or pustular stage, and consist in petechial or larger hemorrhages into the skin beneath the papules, vesicles, or pustules; these hemorrhagic conditions, though of bad omen, are not quite invariably fatal, especially if the hemorrhage is limited in extent and does not occur before the pustular stage.

The primary hemorrhagic form of smallpox is fortunately rare, and its importance consists in its liability to escape diagnosis. If once seen, it forms such a striking clinical picture that it is not likely to be forgotten. The chief difficulty in diagnosis is often felt to be the exclusion of "typhus." Dr. Collie[1] points out that in primary hemorrhagic smallpox "the petechial eruption will be present on the third day, or at the latest on the fourth day, in profusion, which excludes typhus. Moreover, in hemorrhagic smallpox there are blue-black spots, hemorrhage from one or more of the mucous membranes, and bloodclots in the conjunctivæ. In typhus there is nothing of the kind. Hemorrhage from the mucous membrane is almost unknown, and although the conjunctivæ are injected, there is no effusion of blood there."

The absolutely non-characteristic appearance of this form of smallpox, when judged by popular ideas, resulted, as is usual, in the disease escaping notification from the vessel, and leading to some chances of infection in street, train, and cab.

Early information from the General Hospital, however, permitted each thread of such possible infection to be picked up promptly, and no further case has arisen in the city. It is

[1] *Fevers*, by Alex. Collie, M.D. H. K. Lewis.

possible that this form of the disease is not very readily communicable, without prolonged or intimate exposure.

Dr. A. J. Harrison has kindly given me notes of two instructive cases of this form of smallpox which came under his notice in Walsall in 1872, during an epidemic.

The first was in a man, J. S., aged 55, who when seen complained of weakness and severe pain in the lumbar region, which at first appeared simply to indicate a return of previous lumbago. On the following day, however, a purpuric rash began to appear on the arms, legs, body, and thighs, followed on the next day by small bullæ and a few papules on the body, when, without any marked hemorrhage, he rapidly sank, retaining his consciousness almost to the end.

The second case was in a young man, W. W., aged 16, who when first seen complained of great pain in the stomach and back. Pulse 80, respiration irregular, often hurried, tongue very foul, face suffused, conjunctivæ injected; the skin, especially over the trunk, dusky-red. In the evening he was worse: petechiæ appeared on the abdomen and in the loins; the pain was extreme and paroxysmal; there was great restlessness, but little delirium. On the following day the symptoms were more severe, and the patient had had little sleep; no true variolous rash appeared, but the purpuric appearances increased; there was some diarrhœa, the urine clear and plentiful; he took nourishment badly. On the third day the symptoms were again aggravated: bullæ appeared in various parts, slight vesicles on face and chest, but no true variolous eruption; the mind remained clear. About two p.m. he began to pass dark-looking blood *per urethram*, blood appeared in the stools, and blood-stained mucus exuded from the nose and lips. He died at six p.m.

The early diagnosis of this form of smallpox is very important for preventive purposes. A good description will be found in Dr. Collie's work on *Fevers* (Lewis), or in the article by the same writer in Quain's *Dictionary of Medicine*. A very useful article, with well-chosen illustrations from negatives by Dr. Killick Millard, on the general diagnosis of smallpox opportunely appears in the December number of *Treatment* (vol. v., No. 10) from the pen of Dr. Meredith Richards. I have found it possible to get by photography excellent records of the smallpox eruption for teaching purposes, and those accompanying Dr. Meredith Richards' article are both clear and characteristic.

[The results of failure to recognise such a case as this have recently been brought prominently before the public in the case of a medical man who was before the magistrates at the Bow

Street Police Court on January 31st, 1902, who treated a similar case as "purpura hemorrhagica," with the result that twelve persons took smallpox and three died. It is interesting to note that in this case the first rash was an erythema resembling the scarlet fever rash. These preliminary scarlatiniform or morbilliform rashes occasionally precede the true eruption, and should always lead to suspicion of smallpox.—EDITOR.]

